# Griffin Auxiliary Medical Society

**Published:** 1859-04

**Authors:** 


ATLANTA

Medical and Surgical Journal.

VOL. IV.] APRIL, 1859. [No. VIII.

### ORIGINAL COMMUNICATIONS.

### ARTICLE I.

### *Griffin Auxiliary Medical Society.*

In pursuance of a call published in the city papers, addressed to the Physicians of the city of Griffin and the surrounding country, they assembled at Dr. E. F. Knott's Office, on Tuesday, the first of March, 1859.

On motion of Dr. Saunders, Dr. John P. Chatfield was called to the Chair, and Dr. John W. Adams requested to act as Secretary.

The Chairman explained the object of the meeting to be the formation of a Society Auxiliary to the "Medical Society of the State of Georgia."

Dr. E. F. Knott then addressed the meeting, and eloquently portrayed the advantages resulting to the Science of Medicine from a united effort of those engaged in the study and practice of the divine art.

On motion, Drs. J. F. Wright, J. W. Adams, T. M. Darnall, L. J. Greene, and Augustus Wheelus, were appointed a Committee to nominate suitable persons for officers of the Society for the ensuing year.

The Committee reported the names of Dr. E. F. Knott for President, Dr. J. T. Banks for Vice President, Dr. Thos. M. Darnall for Recording Secretary, Dr. D. M. Williams for Corresponding Secretary; which was adopted.

On motion, the Society adjourned to the first Thursday in April. All legitimate practitioners of Medicine desirous of promoting the best interests of the Science, are respectfully invited to attend. T. M. Darnall, M. D., Sec.

---

Griffin, March 2d, 1859.

Dr. E. F. Knott—

*Dear Sir:* We, the undersigned, in behalf of the Medical Society of this place, most respectfully request for publication, a copy of your excellent address, delivered on the first instant, on the importance of forming an Auxiliary Medical Society at this place.

Very Respectfully,

Thos. M. Darnall,

S H. Saunders,

J. H. Connelly, } *Committee.*

---

*To Messrs. T. M. Darnall, S. H. Saunders and J. H. Connelly*—

Gentlemen: Your note requesting manuscript of the address I delivered before Griffin Medical Society, at its Inaugural meeting on first March, has been received. I herewith place the manuscript at your disposal, with many regrets, however, that owing to its having been prepared hastily, and under many engrossing engagements, I did not deem it worthy to be submitted to public scrutiny and criticism; nevertheless, in the fond hope, that it may in some measure, subserve the great interest of the Medical Profession, and the ultimate good of mankind, thereby I yield.

With my best wishes for the body you represent, and yourselves individually, I remain Yours, Professionally,

E. F. Knott.

### ADDRESS.

*Gentlemen of the Medical Profession*—

Allow me to congratulate you that so goodly a number of us have assembled on the present important occasion, and under such auspicious circumstances. Most cordially do I offer you my salutations, and most heartily bid you welcome to these halls, consecrated as they are to the science of medicine, and the investigation of those truths by which alone that science can be sustained. You are now about to engage in an enterprise, absorbing in its interest, and need I tell you that it is one worthy of your best energies; requiring your sincere devotion of heart, and your most unwavering purpose? If you are not prepared to make this acknowledgment—not ready to appreciate the importance of the object before us, in all its magnitude—not willing to make the necessary sacrifices—not free to yield assent to whatever requirements this association may, in its wisdom, see fit to adopt; in a word, if your aspirations are not high, let me, in candor and fidelity, advise you to abandon, not only the purpose immediately in view, but also this noble profession, and seek an occupation which will not involve such sacred and such fearful responsibilities.

It is our happy privilege to live in an age of enterprise and progress. The human mind is actively engaged in scientific investigation, and each day discloses fresh evidences of its triumphs. Truly "the lines are fallen unto us in pleasant places; yea, we have a good heritage." The country in which our lots have been cast, is one from the very nature of her institutions, offering every possible encouragement to industry, in every department of usefulness.—Here, the humble, but deserving physician—the son of poverty it may be—whose means are so restricted as scarcely to afford the necessaries of life, cannot be debarred from the use of that intellect, the priceless gift of a gracious Providence, nor from that full measure of advancement with which its right use is sure to be rewarded. *Mind!* what a noble endowment! Like the sun, it is confined to no particular country or people, but in some degree, at least, is universally diffused.

That we may the better learn to understand our true position, and be led the more to realize that we are each to depend for success, not on adventitious circumstances, but on personal merit and exertions, we have met together to pledge our best efforts to promote the cause of science, and to uphold the standard of our profession. It will not, I imagine, be denied that the practice of medicine does involve most fearful responsibilities. Every conscientious physician must feel that he occupies a sublime position, and whenever he takes a calm survey of his high and accountable station, he is forced to exclaim, "who is sufficient for these things?" It is a position pre-eminently adapted to call forth into exercise, all the noblest attributes of man; his honesty, his integrity, benevolence, sympathy; his utmost activity, industry and enthusiasm; his knowledge, wisdom and discretion. What a noble field of usefulness! What constant exercise does it require for all that is good in a man's head and heart. How it is calculated to stimulate his intellectual powers, and to refine and elevate all his feelings and affections.

Now, gentlemen, if, by such an organization, as we propose now to effect, we shall have our minds constantly "stirred up by way of remembrance," as to the dignity of our profession, the importance of the principles which are to guide us in its practice—if the tendency of our association will be to familiarize us as far as possible, with the hidden mysteries of our science—if, moreover, by our mutual intercourse, a fraternal feeling is fostered, shall we not have occasion to associate in our minds pleasant and grateful thoughts with these halls? The principles to be discussed here from time to time, will be those of truth and of science, and those by which we hope to be enabled to triumph over the charlatans of our day.

Rapid as have been the studies of science, and brilliant as have been the conquests achieved by master-minds in the field of medicine; zealous and laborious as have been its cultivators, there are yet trophies in reserve for the diligent and persevering. If a laudable ambition should actuate us, there yet remain laurels to be won. Let us bear steadily in mind the fact, that in the various departments of medical science, as in philosophy itself in general, there remains "much land yet to be possessed;" much still to be accomplished. As often as we shall meet in these halls, let us stimulate one another to diligence, zeal, whole-souled devotion and perseverance in the profession of our choice. Let us here cultivate a love for all that is pure and holy, and here learn to view with abhorrence whatever may be calculated to degrade this honorable calling. Let us here habituate ourselves to admire and encourage true merit, wherever found, while we are not influenced by wealth or high, though undeserved position, in the world. Let us endeavor to realize the truth, that on the members of the medical profession of this age and generation, must depend its future prospects. If we suffer the standard of the profession to be pulled down from its present lofty and commanding eminence, and its honors to trail in the dust, posterity must reap the disastrous consequences of our conduct. Let us then, gentlemen, give it our hearts' best and purest affections, and resolutely stand up for it; and in the spirit of the dying Lawrence, when we see our high vocation endangered by the prejudices, false notions, and innumerable quackeries of the day, let us cheer each other with the cry, "never give up the ship."

Apart from the empiricisms of the age, there is another cause of anxiety; indeed, incomparably greater anxiety to our minds. The cause of injury to medicine is found in the want of harmony and concert of action among its disciples. By this our profession is deprived of much of its influence and standing. "If a kingdom be divided against itself, that kingdom cannot stand. And if a house be divided against itself, that house cannot stand." In union alone is strength. Dissolve the support which the heavenly bodies afford each other, and throw them into discord, and creation will speedily become a wreck. Disunion is the bone of everything. Nothing can prosper amid civil dissensions. "E pluribus unum" is a motto as necessary to be adopted and acted upon, when engaged in defence of professional truth and character, as in defence of confederated States. But, alas! it is not the principle by which the Medical fraternity has been governed usually. The other professions derive strength and influence from the observance of this precept; but physicians, with equal advantages and opportunities in every other respect, and with a superiority over them in point of numbers, have none of this consolidated authority and influence. Strange to say, so far from this unity of spirit and feeling, how often are they seen opposing and frustrating every man his brother. How often are the bitterest and most malignant feelings cherished towards each other; how often does their conduct towards each other manifest the worst impulses of their nature. Judging from appearances, many think the only way to build up their own reputation, is to demolish that of their brethren, and the only method of recommending their own system of treatment is by censuring that of their rivals. But the mistake is as obvious as it is mischievous. By thus calumniating each other, and detracting from each other's merit, they bring into contempt, as far as their influence extends, the whole profession, and diminish public confidence in its integrity and usefulness. When one physician unjustly condemns another, his tongue is a poisoned weapon to his own good name, and he commits, if I may be allowed the expression, a kind of professional suicide. Thus is medicine brought into disrepute by its own votaries, and persons are led to ridicule and sneer, and taunt us with the trite, but no less deserved scoff "Doctors will differ." "Doctors will differ." Now, is not such a state of things unfortunate?—"These things ought not so to be." Do you inquire how this evil is to be remedied? I answer, by union, gentlemanly harmony and courtesy, and co-operation among the members of the profession. Thus will the strength of all become in some degree the strength of each, and the whole will stand supporting, and supported in a solid phalanx, which shall be impregnable. Who can tell the immensity of good such a union could accomplish, if based upon right principles, and each member actuated by right motives?—Incalculable benefits would thus be conferred upon the profession and mankind generally. There is no insuperable difficulty in the formation of such an association, provided it be attempted with unanimity, vigor and resolution, and with all the light that may be brought to bear upon it. Such a society we have now assembled to organize, for the benefit of ourselves and all in our vicinity. It may be composed of all the respectable physicians residing within its limits. In this way, we hope that light may be thrown upon diseases incident to this section, under the head of Medical statistics; we hope, also, that by this friendly interchange of sentiments and opinions, we will increase in knowledge. The contact of mind with mind will have the effect of quickening our powers, and altogether will serve to bind us by ties of intimate and abiding friendship. Opportunity will thus be furnished for obtaining a more correct and intimate knowledge of each other, and we can regulate the practice by a code of ethics. This society should meet as often as may be practicable, and on each occasion, there should be discussions upon various topics with medicine: annual, semi-annual or such other periodical addresses might be delivered by such persons as may be selected for the purpose, by the society at its regular meetings. You can readily understand the plan; time does not permit me to enter into minute detail. I would most earnestly recommend, gentlemen, and strenuously advise the adoption of a fee bill; such as may be drawn up and presented by a suitable committee, and ratified by the society. It should be expected that every member of the society will adhere rigidly to this. The compensation for professional services rendered by physicians should thus be regulated, and made uniform, and any deviation from the rule, either in the way of exorbitant charges or the other extreme, in order to acquire popularity, ought to be visited by the displeasure and rebuke of the faculty, and if persisted in, the parties should be proclaimed unworthy of all professional fellowship. They are not worthy of being met in consultation, nor any way to be professionally recognized. No physician should ever descend to the dishonorable practice of lowering his fee, with a view to the procurement of favor and employment. Every system of underselling is justly contemned by the noble and magnanimous; it is knavery, neither more nor less; and under-charging in the practice of medicine is no better. A practitioner is most certainly at liberty to remit a fee altogether, but not to injure his profession, and his brethren, and disgrace himself by lowering it.

In the matter of consultation, gentlemen, physicians should invariably act towards each other with the utmost delicacy and the highest sense of honor. Professional etiquette, is by no means to be ignored. In entering the sick room, the attending physician should lead the way, and the case having been explained, the proposition to retire should be made by the consulting physician, who ought to lead in leaving the room. The result of the consultation should be communicated to the sick person or attendants, by the attending physician, in the presence of his colleague, or otherwise, as the case may require. The prescription should not be changed until the next visit, unless circumstances render it peculiarly important. The consulting physician should not visit the patient in the absence of the attending physician, except in case of an especial agreement to that effect. It is not at all proper for any discussion about the disease to be held in the presence of the patient, or his attendants, nor should differences of opinion be divulged. It would only be productive of mischief. If all agreement is impossible, between the two physicians, then this difference of opinion should be made known, and a third physician should be called in, and it is for the patient or his friends to decide the question, who is to withdraw. As soon as all danger is over, the consulting physician should retire, and he is the person from whom the proposition to discontinue, is to emanate. Does an accident happen, or a dangerous case of disease occur suddenly in a family, whose regular physician cannot be obtained, and another is summoned, the latter should yield at once to the family physician, when he arrives. When a physician is himself prostrate upon a bed of sickness, a two-fold duty devolves upon his brother of the profession. Not only is he to render all the service in his power, gratuitously, but to labor for him, taking charge of his patients, and attending all requiring attention in his circle of practice, until the period of his recovery and return to business.

What can be more unfair and dishonorable, than for one member of the profession to attempt to profit by the misfortunes of another? How noble was the conduct of Dr. Mead. He and Dr. Friend were at the head of their profession in London, and rivals in practice. They were also opposed in politics, the latter being a whig, the former a tory. Being suspected of some connection with the Atturbury plot, Dr. Friend was arrested and committed to the Tower, where he remained a State prisoner for nearly six months. In the meantime, Dr. Mead was called into attendance in most of the wealthy families of his rival. All the while he was interesting himself in the behalf of his sorrowing competitor, and laboring most assiduously to procure his release. At length he effected this object, by subjecting himself to heavy responsibilities, as one of the sureties for his future good conduct. Nor did his magnanimity terminate here. He was the first to visit him at his dwelling, after his restoration to freedom. After the most cordial congratulations, he placed on the table a large purse, filled with guineas, saying to his rival, "This, sir, is yours." Dr. Friend replied, "That cannot be, sir, you owe me nothing." He rejoined, "I owe you this and all the contents. The guinea I paid for it, and every guinea in it, I received from your own patients, during your imprisonment. I insist, therefore, on your acceptance of it. I cannot profit by the misfortune of a rival." Actions similar to this, were they common, would indeed confer a lustre upon the profession, infinitely transcending the renown, occasioned by the most exalted abilities. It is contended by some, that physicians, hostile to each other, should never meet in consultation. On the contrary, would it not seem, gentlemen, that the practitioner of physic who cannot so far control his own personal and private animosities, as to meet and confer honestly with any other competent and fair-minded physician, with a view to the preservation of the life, or restoration of the health of a suffering neighbor or friend, must be unworthy of the profession, and only fit to be ejected from it? A skillful physician belongs to the public, and if deserving, is prized among its choicest treasures. He is virtually under oath to exert himself to the utmost to save life and restore health, whenever he possibly can. He is not at liberty to withhold his services, when any opportunity offers, of rendering them with advantage. He is bound by solemn ties, which he cannot, under any ordinary circumstances, violate with impunity. He is in duty bound to make his personal feelings yield to his public character and duties, and to merge the man into the physician. This he will not find at all impossible, if he has had that mental discipline, to which no educated man can be a stranger.

There are yet other courtesies between physicians, which should be strictly observed. The professional attendance upon any member of the family of a brother physician, should always be gratuitous. Of course, if the individual thus obliged, be in circumstances enabling him to do it, he is bound on the principle of reciprocity and good feeling, to make a suitable compensation. A handsome present is an appropriate testimonial of gratitude. When a physician becomes independent, and retires from the profession, it would scarcely be deemed at all fair, for him to attend his neighbors without charge. In this way, he injures the prospects of his younger and less opulent brethren. Instinctive delicacy forbids one physician to visit, as a friend, the patient of another, unless accompanied by the physician in charge. Even then, such a visit should be brief. It would not accord with the rules of strict propriety for him to make a professional inquiry, and he should be careful to retire, leaving the attending physician in the room.

When a family becomes dissatisfied with a physician, while in attendance upon a patient, and without his knowledge calls in another, it is eminently proper for the latter to decline attendance in the case, unless in consultation with the former. In fine, gentlemen, the faculty of medicine should constitute a band of brothers, and they need never expect to attain the highest point of usefulness unless in an associated capacity, recognizing a due regard for the claims of each other. Some may speak slightingly of professional etiquette, but depend upon it, there is such a thing, and a breach of its rules can seldom be made, and the transgressor escape all indignation.

But before I bring to a close these desulatory remarks, I must try to impress you with a due sense of the importance of the object we have immediately in view. I need not add much more on the advantages of Association. In every department you will admit it to be a self evident proposition, that "union is strength"—"United we stand, divided we fall." Unity of sentiment and action is our shield, buckler, and sword. Why are countless hordes of savages and barbarians, forced to quail and succumb before a small band of civilized men? It is for want of well concerted plans and institutions. Failure in efficiency and power among the ancients, notwithstanding their intelligence and enterprise, is, in almost every case, to be attributed to this want of unity. In almost every calling, it is adopted as a rule of faith and action that union is strength. The numerous Medical Societies which have been organized throughout all Christendom, have been the means of blessings unspeakable to the world. They have been useful in making known the improvements in science; in enabling physicians to employ these with more judiciousness in the prevention and alleviation of sickness and suffering, and preservation of life. This statement, if any one were disposed to question it, might be abundantly sustained and corroborated by a reference to what has been done by the various Medical Colleges and Societies of different names, which have been established so numerously in every country where science is at all fostered. And just in proportion to the multiplication of such institutions, and the extent of country over which they have been planted, is the degree of light shed around, and the general good effected. Wherever such Societies have been organized and judiciously conducted, professional enterprise, and science generally, and beneficence have been implanted, fostered, and exercised.

In view of all this, Gentlemen, shall we not congratulate ourselves upon the formation of this association, which I have now the honor to address? Is it not an event of fair and most auspicious promise? Let us endeavor to appreciate its importance. Let there be no half heartedness about it. It requires whole souled energy, in order that its purpose may be carried out. No lasting good can be the fruit of accident only. Away with momentary impulse, hasty conceptions, and immature counsels. "It is good to be *zealously*

affected *always* in a good thing." All improvement is the result only of time and reflection, judicious planning and persevering labor. This is true in respect to the issue of all enterprises whether they be projected by individuals or associations. They must originate in mature deliberation, and be conducted by a well devised system, or else they will certainly fail and be productive of evil rather than of good. To lose sight of its definite and substantial purposes, and the scheme must be such only as is most likely to effect the objects in contemplation.

Medicine divides itself into two departments, the theory and the practice. Our Society has for its object the improvement of ourselves in regard to both. A leading feature here will be inquiry into the causes, character and treatment of diseases as they present themselves to the observation of each member in his round of practice. Each will make known the results of his own experience and observation. The study of the actual seats of disease and of the condition of the organ or organs in which they are located, will afford us matter of absorbing interest, for it is of vital importance in the science. Without a knowledge upon these points, diseases cannot be understood, and therefore cannot be correctly described or rationally and successfully treated. This inquiry involves the study of morbid anatomy, and that imposes the necessity of post-mortem examinations. In cases where any doubt exists, as to the locality, nature and character of a malady, this form of research should never be neglected. Physically speaking, it is the only way to profit by mortality, and to derive from the dead a remedy which may be available for the living. It therefore becomes every member of the profession to do what in him lies, to overcome those prejudices, which naturally, and almost inevitably arise in the public mind against the practical study and investigation in anatomy, and endeavor by all legitimate means, to secure the repeal of those stringent laws which forbid dissections, and thus interpose the greatest obstacle in the way of research, and a revelation of such diseased actions as go on in the living economy, and which are necessarily hidden from our ken. The mere enactment of such a law has tended in no small degree to cherish these prejudices on the part of the community. We have sufficient confidence in the integrity and becoming modesty of our profession, to believe that if such repeal be effected, things would be conducted with due decorum and propriety, and with due deference and regard to the wishes and feelings of the living.

Such, gentlemen, are among my imperfect suggestions, proposed before you at this time. The time for preparation was short, and I have, amid many and engrossing engagements, thrown together these crude and somewhat disconnected reflections. I will only detain you a few moments longer, by offering some words of advice, which I am sure, will be received in the same spirit in which it is given.

In regard to intercourse with each other, and brethren generally, there will devolve upon us duties of no trivial importance. On the manner in which we discharge these relative duties, must depend in a measure, the dignity and position of the profession, and in consequence, the means of accomplishing good among our fellow beings. Truth forces us to the humiliating confession, that medical men are proverbial for discord and division. It is not, however, a necessary accompaniment of the vocation. It is not, that there exists anything in the nature of our pursuits, which attracts into our ranks the factious, rebellious and impracticable. The character which our calling has most unfortunately acquired in this respect, is owing to the promiscuous intercourse which medical men are compelled to have with all classes and grades of society. Hereby they are continually misrepresented to each other, and are often the victims of gossips and mischief-makers. Make it a rule to be ever incredulous of evil reports, and when they reach you in unquestionable form, avail yourselves of the earliest opportunity for an explanation.

Reflect seriously upon the vast importance of the profes sional duties which establish your relation with the public at large. Remember that you have enlisted under the banner of Mercy, and solemnly pledged yourselves to employ every instrumentality which God has placed in your power, to war against death and his ministers. Never, never, will this pledge be redeemed by the cold and heartless exertions which are prompted only by the spirit and the interest of the hireling. Let us "magnify our office." When summoned to the bed of suffering, on which you behold one so dear to many hearts—so necessary to their comfort, support, and happiness—when you feel that every tearful eye is turned imploringly on you for help, and are made conscious that every expression of your countenance is scrutinized for some indication of hope or fear—oh! then how are we made to see the painful responsibilities of our situation! How vast an amount of human sorrow is, under God, influenced by the manner in which the physician's duties are discharged. Sometimes anxiety broods over the whole community, and even the whole nation, as some patient gradually pines away, for whom the sympathies of all are drawn out. How painfully momentous becomes the peculiar position of the physician!

It is cause for thanksgiving that it is our privilege so often to meet in the chamber of sickness and sorrow, drawn thither by her instinctive goodness of heart, the "fairest of creation, last and best of God's works"—woman. She will co-operate with you in your work of mercy. She will appreciate your humane devotion, and reward you with approving smiles.

> "O! woman! in our hours of ease,
> Sometimes uncertain—hard to please.
> When pain and anguish wring the brow,
> A ministering angel thou!"

Seek to deserve her smiles and cultivate her society for its improving and elevating influence. Nothing is so admirably adapted to impart to man that gentle and soothing address which ministers to the mind diseased.

Our profession, too, demands all that moral courage and decision, than which nothing in our nature can display more sublimity. This courage, this *heroism*, is the result of no transient and frenzied excitement like that which often in the turmoil of the battle field impels the warrior upon his foe—it is a calm, deliberate, and sober conviction of duty. This nerves him up when he encounters the grim monster, and enables him to watch and study all the phases he assumes, even at the very moment when his vindictive fury assails his own heart.

Such observations as I have now addressed to you, are no more than can be taught from the unwritten book—the book of nature, and in the school of experience. But written books are not to be neglected by the physician. It is a melancholy fact, and one to which I refer with reluctance, that Southern physicians read far less than they ought to do and far less than they might do. The physician is yet to be seen who cannot, owing to the extent of his practice redeem some portion of time for the perusal of books pertaining to his profession. In half the number of hours that most physicians spend in idleness and unimproving conversation they might accumulate untold treasures of medical knowledge. The want of a library is frequently urged as an apology for this state of things. But this excuse will not hold good, in these days of valuable Medical Journals, Reviews, and such periodicals. By the habitual perusal of one or two of these publications, the most extensive practitioner may do much towards keeping pace with the progress of his profession. Neglect of this duty is neither more nor less than criminal, when such an affluence of means surrounds us. Such writings are repositories of the most important discoveries and improvements; they are rendered by their cheapness accessible to all, and afford a substitute for a library of Standard Works, where the attainment of this is impracticable.

The true art of deriving information from books is too seldom practiced by readers, and is perhaps not generally understood. It is one thing to read and quite another to learn. In order that a book should prove a source of knowledge, it must be *studied.* To study is to analyze, examine, ponder upon, and compare with nature. The physician who does not read in this manner, might almost as well close his book and never look into it. "Reading," says Bacon,

"makes a *full* man; conversation a *ready* man; writing an *exact* man." There is truth in the assertion, that "to study without a pen in hand is but to dream." It is very important that you should have a pen by you, to note down such thoughts and passages as are striking, and to make such extracts as may be most worthy of recollection and best suited to the attainment of your object in view, or to the illustration of any point you may be investigating. In doing this, the attention is at once excited and fixed. The matter is more fully comprehended and digested, and retained longer in the memory. Such a digest or analysis may be prepared with ease, and contributed to enrich the pages of some periodical, and thus you might benefit the public as well as yourself, or, at least, in this way add much interest to our meetings in this organized capacity.

But the noblest end at which Medical Societies can aim, and for the attainment of which every nerve should be strained, is yet to be expressly declared, though it has been intimated in our train of remarks already, and follows inferentially from what we have said. It is the purification of our profession from all unworthy motives; its purgation from all that is base, sordid, and sinister; its general improvement in morality and honor, and its maintenance in dignity and worth. It is a lofty and sublime calling, the fairest and most efficient handmaid of benevolence and philanthropy, and not a trickish, grovelling, money-making scheme of barter and traffic. Its legitimate and laudable end is to minister to humanity, and not to personal cupidity, and selfishness; to save life, restore health relieve suffering, and alleviate distresses of friends; not to gratify ones acquisitiveness, fill the coffers of the avaricious, the covetous and the uncharitable. The true hearted physician, when summoned to the scene of sorrow and anguish, thinks of the surest and most speedy way of doing good, the credit he may do his profession, and not what amount of pecuniary profit will accrue from his labor. He forgets himself, is alike insensible to danger, discomfort and selfish considerations; so engrossed is he with his pressing duties in times of emergency and peril. The mere hireling who enters this profession only for the sake of the "loaves and fishes," and is not actuated by sympathy for suffering humanity, and by a desire to add new lustre to the vocation, will never rise to an enviable distinction. He may prove to be a successful trader in it, but nothing more. He will never be decorated with its honors while living, nor have his memory embalmed by either its praises or regrets when dead. Like the ingrate who, heartlessly indifferent to his country, and whom the poet has justly consigned to infamy,—the sordid and penurious trafficker in Medicine.

> "Living, shall forfeit fair renown
> And doubly dying shall go down
> To the vile earth from whence he sprung,
> Unwept, unhonored and unsung."

Gentlemen, opportunity has not been wanting to each of you to test the principles I have now sought to inculcate upon you. Should your "pure minds" by these means "be stirred up by way of remembrance," my object is attained. These principles are just and true, and you have found their application productive of safety to others and successful reputation to yourselves.

And now I conclude with the last but not least precept, and would it might be engraved upon the tablet of each heart as "with a pen of iron, and with the point of a diamond!" While engaged in the contest for which you have armed yourselves, with the grand enemy of our race, forget not to invoke the aid of that great Ally who "hath vanquished death and the grave," and can enable us to conquer also. Thus will your career upon earth be useful, and you will have the gratifying consciousness that your fellow-men have been made the better and the happier by your existence in the world. In pronouncing this may my voice be prophetic, and may future years, which shall find this voice silent, and only "Hic Jacet" inscribed on the tomb of its possessor, find you in the enjoyment of health, happiness, and a well-earned good name, and when you shall in turn be summoned to render an account of your stewardship, may the welcome plaudit of the "good and faithful" servant be yours.

